# Early identification of cancer-related malnutrition in patients with colorectal cancer before and after surgery: a literature review

**DOI:** 10.1007/s00520-022-07230-z

**Published:** 2022-06-23

**Authors:** Elke Wimmer, Agnes Glaus

**Affiliations:** 1 Oncological Care, Careum University of Applied Sciences Health, Zurich, Switzerland; 2 Oncology Nursing and Science, Tumor and Breast Centre ZeTuP, CEO Foundation SONK (Foundation for Educational Activities in Oncology), St. Gallen, Switzerland

**Keywords:** Malnutrition, Colorectal cancer, Nutrition screening tool, Nutrition assessment, Applicability of tools

## Abstract

**Purpose:**

The aim of this literature review is to provide a comprehensive overview of methods for early identification of cancer-related malnutrition and/or risk of malnutrition in patients with colorectal cancer. The focus is also on applicability and feasibility of the use of nutritional tools in oncology clinical practice.

**Methods:**

The literature search was conducted from November to December 2020 in the health science databases by two independent persons. Inclusion criteria were English and German language and articles from 2010 to 2020. Data analysis was carried out through a structured procedure. The research questions guided the literature review.

**Results:**

After removing duplicates and screening titles and abstracts, a total of 35 studies were identified as suitable publications and further analyzed. Eventually, nine original studies, with a total of 926 patients with colorectal cancer before or before and after surgery, addressed assessment measures for early identification of the risk or presence of malnutrition. The following types of nutritional assessment have been described: nutritional anthropometric measurements, laboratory chemistry diagnostics for malnutrition, and several validated nutritional screening and assessment tools. The nutritional tools demonstrate differences in terms of application and content. None of the reviewed studies was a randomized trial. There is little scientific evidence to underpin their specific application in identifying early cancer-related malnutrition in patients with colorectal cancer.

**Conclusion:**

The early assessment of nutritional status in this patient group seems to lack evidence-based standardization in oncology clinical practice. Different groups of health professionals are involved; however, studies do not describe standardized roles. Physical activity as part of nutritional screening is not yet included in the analyzed screening tools.

## Introduction

Colorectal carcinoma (CRC) incidence increases after the age of 50 years. An estimate of the European Union in 2020 reveals that colorectal cancer accounts for 12.7% of all new cancer cases and for 12.4% of all cancer deaths. Thus, it represents the second most common cancer, after breast cancer, and the second most common cause of cancer death, after lung cancer [1]. The prognostic assumption that the incidence of colorectal carcinoma is decreasing, due to comprehensive screening programs, should not hide the fact that there is still a high risk of developing colorectal carcinoma. Besides a genetic disposition, risk factors such as tobacco and alcohol consumption, obesity, lack of exercise, and a low-fiber diet are discussed as causative factors [2].

The high incidence of colon cancer and the frequent presence of concurrent cancer-related malnutrition in these patients [3] was the main reason to focus on this patient population in this review. To achieve the highest possible homogeneity, the focus was on pre- and postoperative patients as multiple treatment modalities and stages of the disease may have made comparison difficult. Eventually, identifying early malnutrition in these patients pre- and postoperatively may have the potential to provide effective, therapeutic interventions. Patients with cancer have an increased risk of malnutrition due to both, disease and treatment. Depending on tumor type, stage, and age, the prevalence of malnutrition varies from 20% to more than 70% [4]. Nutrition assessment is needed in order to identify and treat malnutrition at an early stage, as it can have a negative impact on treatment outcomes in terms of postoperative complications, chemotherapy efficacy, and tolerability [5, 6].

Despite this scientifically verified knowledge, summarized in the European Society for Parenteral and Enteral Nutrition (ESPEN) guidelines on nutrition in cancer patients [2], there is a need for improvement, as cancer-related risk of malnutrition is still too often not assessed [7] and, therefore not noticed early enough by health professionals, is overlooked or not treated adequately [4]. A recent survey found that up to 43% of clinicians (dietetic, nursing, medical) have limited or no confidence in their ability to detect malnutrition and sarcopenia [8]. The assessment options for the early identification of cancer-related malnutrition are diverse and require different professional skills as well as interprofessional collaboration. Therefore, an interprofessional approach to the early assessment is undoubtedly essential in order to diagnose the presence of malnutrition early so that the potential downward spiral can be prevented or stopped. Uncertainties or inexperience on the part of the professionals may be one of the reasons why routine nutritional screening and assessment in oncology patients is not yet widespread [9]. Challenging is the fact that malnutrition may not be visible at first sight, because overweight and obese cancer patients can also be malnourished, and this could have a negative effect on the course of the disease. Sometimes, patients are happy initially to have lost weight without suspecting that this could be a serious sign of disease. If malnutrition is already present and remains undetected, it is often associated with a negative disease prognosis [10, 11].

Several studies have looked at different types of assessment for early identification of malnutrition. It was not possible to identify studies summarizing the current state of knowledge and practice in assessing cancer-related malnutrition and/or the risk in patients with colorectal cancer before and after surgery. This indicates that research and practice in early identification of malnutrition in clinical practice may still be neglected.

## Purpose and research question

The aim of this review is to provide a comprehensive overview of methods for early identification of cancer-related malnutrition and/or risk of malnutrition in patients with colorectal cancer, as described in the current literature. The focus is also on applicability and feasibility in oncology clinical practice. Following research questions are addressed: What type of assessment was performed in currently available studies analyzing early identification of cancer-related malnutrition and/or the risk of malnutrition in patients with colorectal cancer before and after surgery? Which available nutrition tools are described and proposed to provide the most accurate indication of malnutrition and/or risk of malnutrition in these patients?

## Methods

### Search strategy

The first literature search was conducted from November to December 2020 in the databases Pubmed, Cochrane library, CINAHL, and Embase by two independent persons. In addition, a hand search was conducted for further publications. Inclusion criteria were English and German language and articles from 2010 to 2020. The following keywords were selected in connection with mesh terms: malnutrition, nutrition risk, underfeeding, colorectal cancer, screening, assessment, survey, and questionnaires. Studies were excluded if they involved patients undergoing or planning to undergo chemotherapy, if they focused mainly on biometric data, preoperative nutritional markers, or had an exclusive focus on economic issues.

## Results

**Fig. 1:**
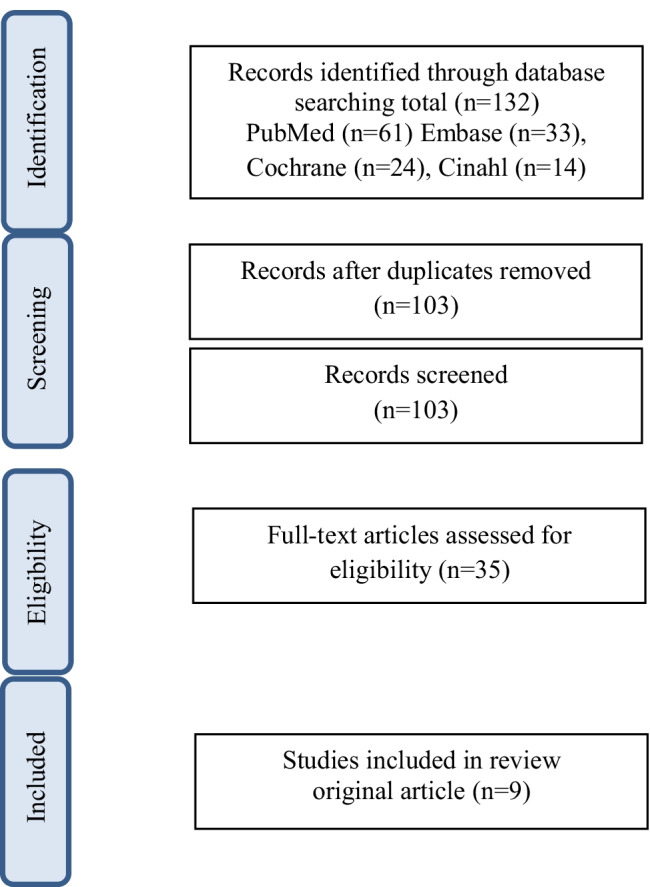
Literature search process

The literature search resulted in 132 articles. After removing duplicates and screening titles and abstracts, a total of 35 studies were available as full text publications. Eventually, nine original studies were analyzed to answer the research questions (Fig. 1). All original studies with a total of 926 patients with colorectal cancer evaluated assessment measures for early identification of the risk or presence of malnutrition. Two studies selected patients in the outpatient setting [12, 13], and six focused on hospitalized patients [9, 14–18]; in one of the studies, the setting was not explicitly described [10]. All articles focused on the nutritional status of patients in a preoperative situation; six of these studies assessed both the preoperative nutritional situation and compared it to the postoperative nutritional status [15, 17] or with a postoperative predictor [9, 14, 16, 18] (see Table 2).

### Nutritional anthropometric measurements, laboratory chemistry diagnostics

In the reviewed studies, a wide variety of assessment methods to measure nutritional status was used. Nutritional anthropometric measurements were performed in all studies with varying intensity. These included measurements of body mass index, body weight, height, phase angle, body composition computer tomography (CT) images, abdominal circumference, waist circumference, waist-to-height ratio, waist-to-hip ratio, upper arm circumference, upper arm muscle circumference, and triceps skinfold. In six of the studies, laboratory chemistry diagnostics were additionally collected and mainly included inflammatory markers, serum albumin, and prealbumin (see Table 2). One study also looked at selected signs and symptoms such as dysphagia, mucositis, odynophagia, dental prosthesis inadequacy, xerostomia, and also alcohol consumption [12]. In order to identify other influences on nutritional status, diabetes mellitus [18] smoking behavior [9, 12, 18], and blood transfusion [9] were explicitly mentioned as a parameter in a few studies. Physical activity behavior [12] and hand grip strength were included in a few studies [9, 13].

### Content and focus of nutritional screening and assessment tools

In all studies, nutritional screening or assessment tools were used to assess and evaluate the nutritional status of patients with colorectal cancer before and after surgery. These are summarized in Table 1.

**Table 1:** Content and focus of nutritional screening- and assessment tools of reviewed studies

	Nutritional screening tools	Nutritional assessment tools
Malnutrition Universal Screening Tool (MUST) [13, 14, 17]	Recent unintentional weight loss, BMI, acute disease severity	
Nutritional Risk Index (NRI) [17]	Calculating a NRI (= 1.519 × serum albumin (g/L) + 41.7 × (present weight/usual weight)	
Nutrition Risk Scale 2002 (NRS 2002) [18]	Recent unintentional weight loss, BMI, severity of disease, age > 70 years, impaired general condition	
Mini Nutritional Assessment (MNA) [10] 18 items divided into four sections		*Anthropometric assessment*: weight, height and loss of appetite *General assessment*: mobility, lifestyle, number of current medications, presence of emotional stress or depression *Nutritional assessment*: number of full meals eaten daily, variety of food and fluid intake *Subjective assessment*: self-perception of health, physical appearance and nutritional status
Subjective Global Assessment Tool (SGA) [12, 13, 16, 17] two sections		*Anamnesis*: weight change, gastrointestinal symptoms, exercise capacity, underlying condition *Clinical examination* (on the basis of the examiner’s assessment of the patient’s nutritional status): subcutaneous fat, muscle mass, edema
Patient Generated Subjective Global Assessment (PG-SGA) [9, 15] two sections		*Self-assessment*: weight history, food intake, nutrition impact symptoms, activity, and functional capacity *external assessment*: diagnosis, disease stage, age, components of metabolic demands (sepsis, neutropenic or tumor fever, corticosteroids), and physical examination

### Psychometric properties of screening and assessment tools

The nutritional screening instrument MUST was used in three of the studies to assess nutritional status [13, 14, 17]. One study indicated that MUST is an independent predictor of preoperative colorectal patients’ risk in relation to hospital length of stay and Kaplan–Meier survival curve [14]. One study used MUST and SGA in the same patients and compared results of the nutritional status of these colorectal cancer patients. With minor differences, results were congruent [13]. One of these studies measured the sensitivity and specificity of the nutrition assessment tools MUST and NRI and used the reference standard SGA, which resulted in a high sensitivity of 96% in the MUST tool and of 95% in the NRI tool and a lower specificity of 63% in the MUST tool and of 75% in the NRI [16]. Two other researchers decided to choose the SGA tool and justified this on the basis of its ease of use and high correlation with nutritional anthropometric and biochemical parameters [12, 16]. The NRS 2002 was selected in a study because the validity of the NRS 2002 had been confirmed as an effective predictor in previous studies, usually for preoperative nutritional risk assessment [17]. One study used MNA for nutritional assessment in a study with the rationale of multiple validation of the instrument^9^. In two studies, the reasons for the use of the PG-SGA tool was its good validity [9, 15]. The scored PG-SGA presented a sensitivity of 98% and a specificity of 82%. The positive predictive value was 95%, and the negative predictive value was 93% [19].

### Applicability and feasibility of the use of these nutritional tools in clinical practice

The authors of some of publications commented on aspects of feasibility and applicability of the nutrition screening or assessment for the pre- and postoperative phase in colorectal cancer patients. In one study, feasibility was attributed to MUST, as it is less extensive and easy to use, although a more accurate test result was achieved with SGA, but it is described to require more training and takes two to three times longer for its use than MUST [17]. The PGA tool is specifically suitable for cancer patients as it was developed for this population [9, 15], which was further developed on the basis of the SGA by Ottery (1994) [20]. This nutritional assessment tool is also described as a feasible, easy to use, and cost effective tool [9]. After comparing the SGA with the MUST nutrition tools, it was concluded that both can be used for preoperative assessment in colorectal cancer patients, as they did not show major differences [13]. Three of the nine studies used retrospective data. The nutritional screening tools MUST [14], NRS [18], and assessment tool SGA [16] are already implemented in current oncology clinical practice.

## Discussion

### Nutritional anthropometric measurements, laboratory chemistry diagnostics

A variety of nutritional anthropometric measurements have been performed in all studies, but not all researchers have undertaken measurement of muscle mass (see Table 2). Body composition analysis including fat, protein, minerals, and body water provides complimentary and more accurate information about the nutritional status than just weight or BMI. Changes in fat-muscle mass and body fat percentage were described in these studies. In practice, this would mean that in addition to the parameters of weight or BMI, body composition would be essential for the early identification of malnutrition. Overweight and obesity are relevant risk factors for people suffering from cancer such as colon cancer. Weight loss in these patients is often overlooked or not identified early enough, because fat may hide the loss of muscle mass. Comparison between the original weight and weight at diagnosis is therefore important to identify malnutrition early [2, 15]. As shown in Table 2, a number of the anthropometric measures are in fact included in most available instruments. It remains unclear whether these were assessed twice or whether they have been adopted from the nutrition instruments.

**Table 2:** Summary of key elements of nutritional measurement in patients with colorectal cancer in reviewed studies

	Almasaudi et al. 2019 [14]	Barbosa et al. 2014 [12]	Burden 2010 [13]	Daniele et al. 2017 [10]	Lopes et al. 2013 [15]	Mauricio et al. 2018 [9]	Nishiyama et al. 2018 [16]	Tu et al. 2012 [17]	Wang et al. 2020 [18]
Setting	Inpatients pre- and postoperative	Outpatients preoperative	Outpatients preoperative	Inpatients preoperative	Inpatients pre- and postoperative	Inpatients pre- and postoperative	Inpatients pre-and postoperative	Inpatients pre- and postoperative	Inpatients pre-and postoperative
Design	Retrospective data analysis	Cross-sectional study	Cross-sectional study	Cross-sectional study	Observational study	Prospective cohort study	Retrospective + analytical cross-sectional study	Cross-sectional study	Retrospective data analysis
Health professions involved to assess nutrition status	Clinical nursing staff and physicians	Nutrition specialist	Not available	Not available	Health professionals	Two trained evaluators	Trained nutritionist	Not available	Clinicians
Screening tool*	*MUST* (*n* = 363) Medium risk (9%) High risk (12%)		*MUST* (*n* = 81) Medium risk (24%) High risk (22%)					*MUST* (*n* = 45) Medium + high (44%) *NRI* (*n* = 45) (pre-/postoperative) Moderately (pre-op 42%, post-op 71%) Severely (pre-op 4%, post-op 26%)	*NRS 2002* (*n* = 120) With nutritional risk ≥ 3 (60%)
Assessment tool*		*SGA* (*n* = 66) Mild/moderate risk of malnutrition (26%) Severe risk of malnutrition (11%)	*SGA* (*n* = 85) Moderate risk of malnutrition (25%) Severe risk of malnutrition (17%)	*MNA* (*n* = 78) Risk of malnutrition (46%) Malnourished (24%)	*PG-SGA* ( *n* = 50) Before/after surgery moderate malnutrition (8% /2%) Severe malnutrition (0%/ 2%)	*PG-SGA* (*n* = 84) Moderately + severely malnourished (52%)	*SGA* (*n* = 40) Moderately + severely malnourished (43%)	*SGA* (*n* = 45) Moderately + severely malnourished (36%)	
Nutritional anthropometric measurements*	*BMI*, body composition: CT images: level of the third lumbar vertebra	BMI, *percentage of weight loss,* arm circumference, triceps skinfold, arm muscle area, mid-arm circumference	*Weight*, *height*, body composition, dynamometry bioelectrical impedance analysis, grip strength	*BMI*, waist-, *calf-, mid upper arm circumference,* fat free mass, fat mass, measuring 4 folds: biceps, triceps, subscapularis, supra iliac by picometer FAT-1	*BMI, % of weight loss, triceps skinfold,* mean of three arm circumference measurements fat free mass, fat mass, electric bioelectrical impedance analysis	*BMI, % of weight loss,* phase angle, muscle mass by computerized tomography; combination of muscle mass and handgrip strength Bioelectrical impedance analysis	*Weight*, height, bioelectrical impedance analysis	*BMI,* Body weight 3 months before hospitalization and at admission triceps skinfold thickness mid upper arm circumference	*BMI*
Laboratory chemistry diagnostics	C-reactive protein, serum albumin	Not available	Not available	Inflammatory parameters (no specification)	Not available	C reactive protein, serum albumin	Serum albumin, hematocrit	C-reactive protein, serum albumin and prealbumin, liver, iron transporter protein, zinc, hemoglobin, lymphocyte	Preoperative: prealbumin, transferrin, total cholesterol, blood glucose, hemoglobin, red blood cell and lymphocyte counts

MUST, Malnutrition Universal Screening Tool; NRI, Nutritional Risk Index; NRS, Nutrition Risk Scale; MNA, Mini Nutritional Assessment; SGA, Subjective Global Assessment; PG-SGA, Patient Generated Subjective Global Assessment; details describe in Table 1: Nutritional screening and assessment tools

^*^Italics: assessment tools include the anthropometric measurements; they are additionally mentioned in the studies

Laboratory chemical diagnostics with regard to malnutrition have been described in six of the reviewed studies. In two of these studies [17, 18] laboratory chemistry diagnostics were performed in more details (see Table 2). It can be argued that single laboratory parameters seldomly provide an indication of cancer-related malnutrition and it remains unclear whether they are enough relevant and cost-effective and whether these, as well as anthropometric measurements, do provide enough scientific evidence for its routine use.

### The potential influence of physical activity in cancer-related malnutrition

The reasons described for cancer-related malnutrition and loss of muscle mass are, in addition to reduced food intake and reduced physical activity, also caused by cancer itself through catabolic metabolic derangements [2]. Nutrition screening [14, 18] or nutrition assessment [9, 10, 12, 15, 16] or both methods [13, 17] are used in the reviewed studies. It is evident that screening instruments are short and concise compared to assessment instruments, as these mainly identify high-risk patients. The fact that all the nutrition screening instruments do not assess physical activity (MUST, NRI, NRS) can be seen as subject to debate. The lack of physical activity items in the nutritional screening tools may be due to the fact that the causative relationship to cancer-related malnutrition was not known at the time of the questionnaire development. Assessment of physical activities could present a potential for structured rehabilitation with the aim of preventing loss of muscle mass and to build up strength again. Therefore, the lack of physical activity items within internationally recognized tools needs to be considered when assessing cancer-related malnutrition. In the meantime, a separate physical activity assessment may be used additionally.

### Psychometric properties of nutritional screening and assessment tools

Validity criteria of the nutrition instruments used in the studies vary, and descriptions of validity or reliability are scarce. The fact that only few studies were suitable for analysis in this review and that a variety of different instruments was used shows the difficulty to underpin clinical practice with evidence-based knowledge. This raises the question of whether validity of instruments is considered satisfactorily in clinical practice. Clinical practice requires valid and reliable instruments. It seems that the studies analyzed did show these requirements. The PG-SGA is the only instrument available that has been developed and tested specifically for cancer patients with satisfying psychometric properties [19].

### Applicability and feasibility in clinical practice

Which screening assessment tool is to be recommended for daily practice to identify cancer-related malnutrition or the risk of malnutrition in the patient population under consideration? Applicability and feasibility present very relevant factors as time and professional resources in clinical practice are limited. The use of MUST is recommended by Tu (2012) because of its easy use, knowing that its validity is lower than SGA. Authors argue that it requires little training and less time, while SGA requires intensive training and takes 2 to 3 times longer [17]. Burden (2010) described the use of MUST and SGA in the same patients which lead to almost identical results in preoperative colorectal cancer patients [13]. These two statements are challenging because the short measurement instrument provided more or less the same results as the more extended assessment tool. This needs to be cautioned because the study included small patient numbers. In the ideal world, a more in-depth nutrition assessment tool is recommended, if there is a malnutrition or a risk of malnutrition diagnosed by a screening tool. Comparing a nutrition screening tool with a nutrition assessment tool does not make sense, as assessment always follows screening. It can of course be considered whether screening is sufficient for the early identification of cancer related malnutrition in clinical practice. Eventually, the authors of this manuscript believe that a short screening is always better than no screening and they acknowledge that clinical circumstances may determine clinical practice more than appropriate, evidence-based assessment tools. Professionals should at least strive for standardizing screening for unintentional weight loss and loss of muscle mass but may not be in the privileged position to apply time-consuming assessment instruments. Nevertheless, raising the awareness for the early identification of malnutrition remains a major challenge for medical professionals anyway.

PG-SGA, the only nutritional assessment tool developed and tested [3] for cancer patients, described by Mauricio et al. (2018) [9] and Lopes et al. (2013) [9, 15], is evaluated as the gold standard for early identification of cancer-related malnutrition due to its low cost and wide applicability [9]. Furthermore, it includes a self-assessment and an external assessment. Patient reported outcomes in nutrition self-assessment may reflect the reality even better than the care giver assessment. The self-assessment part is known as the PG-SGA short form, an independent screening tool, and has been confirmed by Abbott et al. (2016) as a valid instrument in chemotherapy outpatients [21]. In clinical practice, this may be an acceptable tool to screen for malnutrition, as it is validated for cancer patients and is short and easy to use.

### Limitations of the review

Seven out of nine studies have a sample size of less than 100 patients, and none of them is a randomized trial. The studies are heterogeneous regarding objectives, methods, patient categories, and content of the screening and assessment tools.

## Conclusions

The early assessment of malnutrition in this patient group seems to lack evidence-based standardization in oncology clinical practice—and there are many reasons for this. There are nutritional instruments ready to use with different content and focus; however, only one has been developed for patients with cancer, such as colorectal cancer. Although guidelines recommend the application of screening and assessment, resources seem to be scarse. A variety of health professionals are involved, but the studies do not describe standardization of professional roles. Physical activity is not included in the reviewed nutritional screening tools and therefore needs to be addressed in further research. In addition, larger controlled studies could potentially provide practice-relevant evidence on how to identify early cancer-related malnutrition in a practical way, as this has not yet been done in a satisfactory way.

## Data Availability

Not applicable.
